# Exploring the key communicator role of exosomes in cancer microenvironment through proteomics

**DOI:** 10.1186/s12953-019-0154-z

**Published:** 2019-10-29

**Authors:** HuiSu Kim, Dong Wook Kim, Je-Yoel Cho

**Affiliations:** 1 Department of Biochemistry, BK21 Plus and Research Institute for Veterinary Science, School of Veterinary Medicine, Seoul National University, Seoul, South Korea; 2 Department of Biochemistry, College of Veterinary Medicine, Seoul National University, 1 Gwanak-ro, Gwanak-gu, Seoul, 08826 Korea

## Abstract

There have been many attempts to fully understand the mechanism of cancer behavior. Yet, how cancers develop and metastasize still remain elusive. Emerging concepts of cancer biology in recent years have focused on the communication of cancer with its microenvironment, since cancer cannot grow and live alone. Cancer needs to communicate with other cells for survival, and thus they secrete various messengers, including exosomes that contain many proteins, miRNAs, mRNAs, etc., for construction of the tumor microenvironment. Moreover, these intercellular communications between cancer and its microenvironment, including stromal cells or distant cells, can promote tumor growth, metastasis, and escape from immune surveillance. In this review, we summarized the role of proteins in the exosome as communicators between cancer and its microenvironment. Consequently, we present cancer specific exosome proteins and their unique roles in the interaction between cancer and its microenvironment. Clinically, these exosomes might provide useful biomarkers for cancer diagnosis and therapeutic tools for cancer treatment.

## Background

**Fig. 1:**
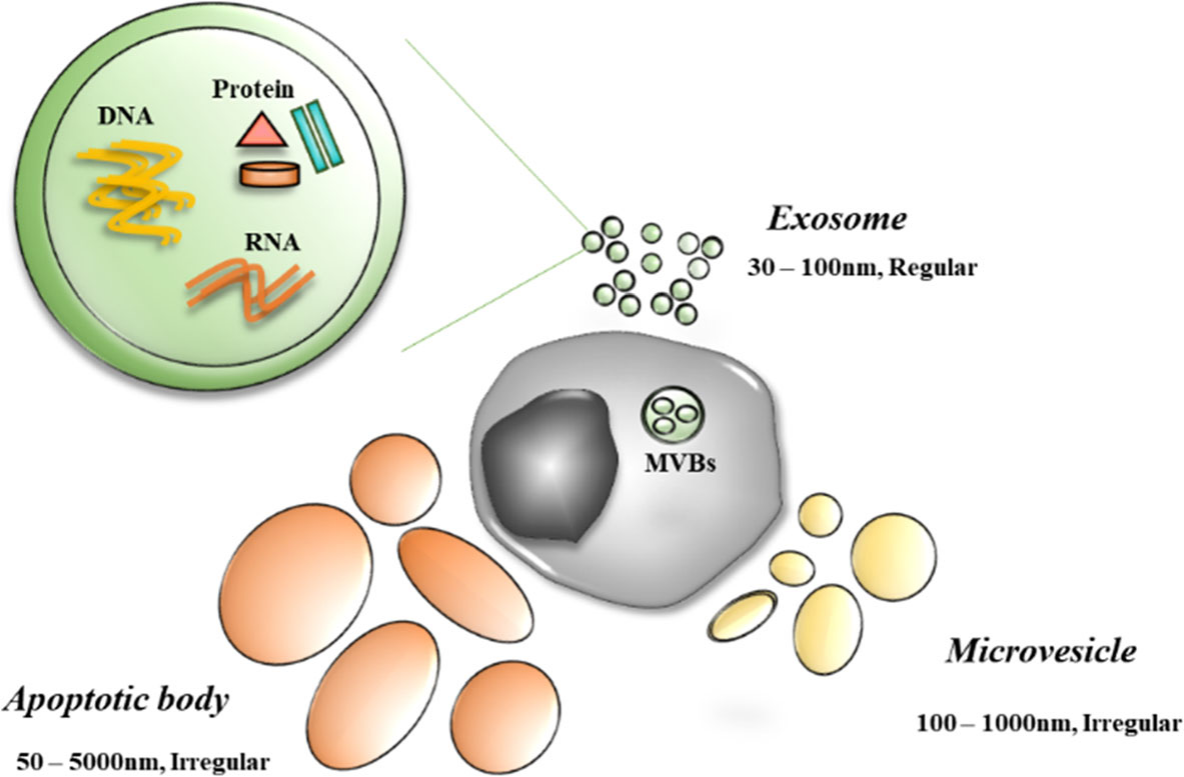
Schematic description of the extracellular vesicles, Exosomes are smallest extracellular vesicles (30-100nm) secreted from endosomes. Microvesicles are small vesicles (50-1,000nm), and apoptotic bodies are largest extracellular vesicles, both are originated from cell membrane

Cell release diverse types of extracellular vesicles; apoptotic bodies whose sizes are 50 to 5,000nm with their irregular lipid bilayers, as well as microvesicles whose size 50 to 1,000nm is smaller than apoptotic bodies but also has an irregular shape. Exosomes are 30-100nm in diameter and contain DNA, miRNA, mRNA, lncRNA, proteins, etc. within their lipid bilayer membrane [1–5] (Fig. 1). Apoptotic bodies and microvesicles are originated from cell membrane surface. Exosomes are smallest extracellular vesicles and originating from endosomes [6]. Exosomes are secreted by various cell types and conditions [7]. After being released from the donor cells the, exosomes travels through the blood and other body fluids. While traveling through the body, exosomes enter the recipient cells through membrane fusion and induce transcriptional and, even more abundantly, translational changes [8–10]. Tumor cells however secrete more exosomes than normal cells and these cancer-derived exosomes are involved in tumorigenesis, metastasis and forming the tumor microenvironment [11]. Recently, many researches have revealed that the exosome is a mediator of cell to cell communication and can be a good candidate for a liquid biopsy biomarker [12–16]. There have been analyses of breast cancer-derived exosomal proteins by liquid chromatography-mass spectrometry (LC-ms/ms), which revealed that the exosome contains a variety of proteins, for example, enzymes, membrane proteins, heat shock proteins, and even transcription factors. This review discusses cancer-derived exosomal proteins and their roles in the interaction with tumor microenvironment.

## Exosome isolation and protein digestion for proteomics

After many research studies proved that exosomes play a role in cell to cell communication through proteins, the interest in exosomes continued growing. However, the method of exosome isolation and analysis is still debated [8]. High yield and purity can not only enhance quality but also help us to understand the exosome’s role in specific conditions. Here, we will discuss exosome isolation methods and digestion methods of exosomal proteins from plasma/serum and cells.

Various exosome isolation methods have been developed [8, 17, 18]. Many of these methods can be categorized into three main categories according to the characteristics of the exosome; density, size, and immunoaffinity. First, sorting exosome by density is the most common method and utilizes differential centrifugation by varying the g force. Shortly, this is started with centrifuging at 300-500g to remove cells, accelerating the speed to 2,000-20,000g to remove cellular debris, and finally speeding up to 100,000-200,000g for the exosome isolation. Using this method, researchers can get exosomes in the pellet. However, isolation takes a long time and requires a lot of input. The biggest drawback is relatively low efficiency and poor recovery. Recently, commercial precipitation reagents have been developed. Using a precipitate for exosome isolation has a higher yield than using an ultracentrifuge, but lower quality since the precipitate can lead to the precipitation of proteins. Second, using the smaller than 200nm size characteristic of the exosome allows it to be separated by filtration and size exclusion chromatography. Filtration and size exclusion chromatography can filter out the cell membrane, sub-cellular fraction and anything that has a bigger size than the exosome. To increase efficiency and purity, many researchers use a combined method, such as filtration and ultracentrifuge, or filtration and precipitate reagents. Muller et al suggested that this combined method is better than using only one method [19]. Lastly, the immunoaffinity for isolation method uses antibodies to capture exosomal proteins. The common proteins isolated by immunoaffinity are tetraspanins such as CD9, CD63, and CD81.

**Fig. 2:**
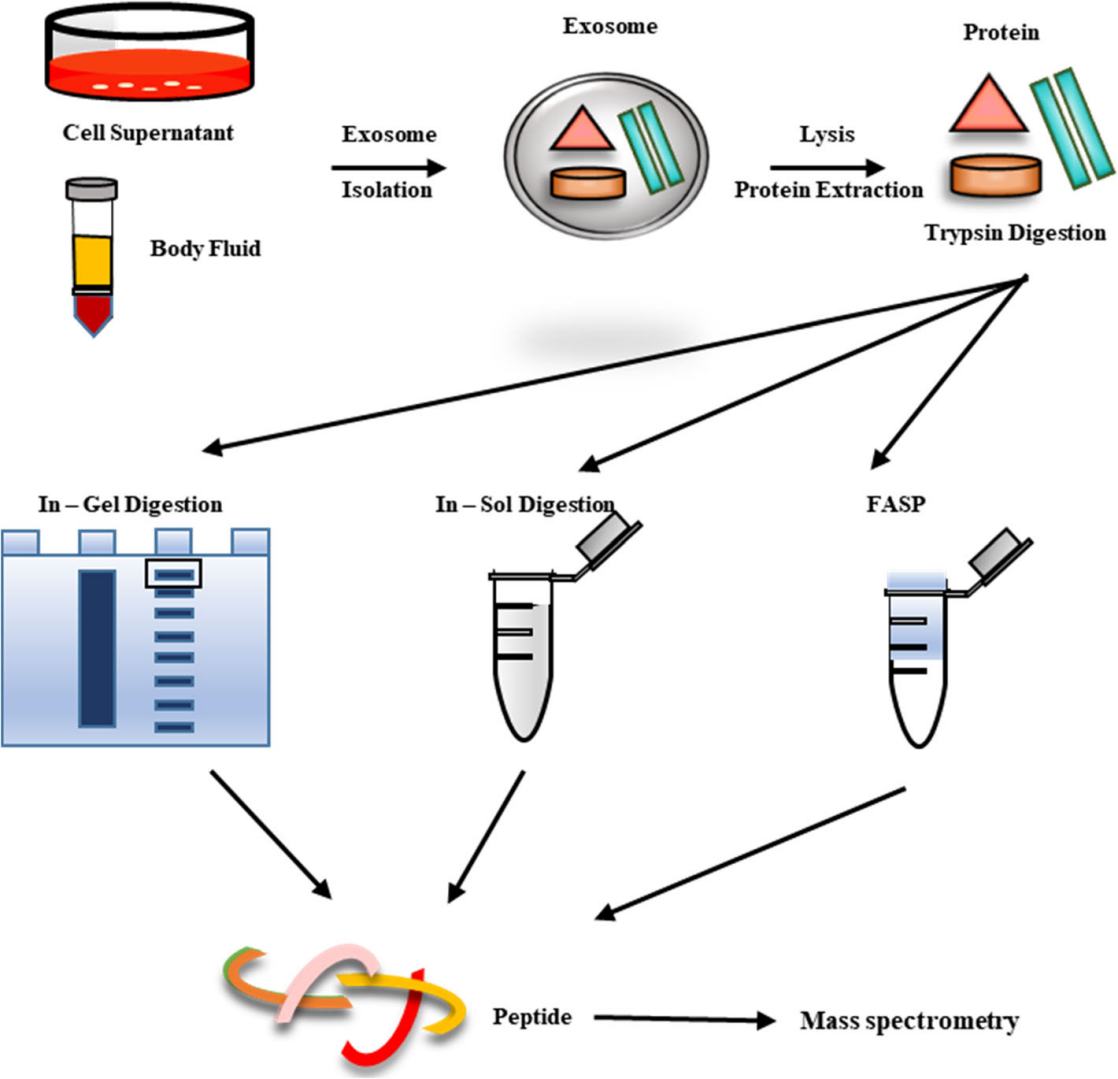
Summarization of exosomal protein digestion methods. Exosomes from cell supernatant and body fluid are digested by (1) In-gel digestion (2) In-sol digestion and (3) FASP methods

After isolation of the exosome, we must lyse the lipid bilayer membrane and digest proteins to peptides for mass spectrometry (MS) analysis. Here, we summarized 3 protein digestion methods; In-gel digestion, In-sol digestion, and Filter Aided Sample Preparation (FASP) (Fig. 2) [20]. First, in In-gel digestion, lysed exosomal proteins are resolved on a polyacrylamide gel and visualized using Coomassie brilliant blue or other staining reagents. The gel is then sliced to a 1mm size and destained by ammonium bicarbonate. The next steps are reduction, alkylation, and digestion. Peptides go through the process of enrichment and cleanup. Then, dried peptides are resuspended and injected into LC-ms/ms [21]. Second, for In-sol digestion, lysed exosomes are kept in an aqueous state. Exosomal proteins sequentially undergo reduction, alkylation, and digestion in the aqueous state. Like the in-gel method, peptides are then enriched and cleaned up before being injected into LC-ms/ms. Lastly, in the FASP method, all of the above-mentioned processes are processed on Microcon 30k centrifugal ultrafiltration units. Lysed exosomes are loaded onto the filter and discard the elute after centrifugation. Reduction, alkylation and digestion are all processed on the filter. Overall, each method has its advantages and disadvantages. Here, we summarized methodological properties in Table 1 [21, 22].

**Table 1:** Advantages and Disadvantages of proteomic digestion techniques

Digestion Method	Advantages	Disadvantages
In-gel Digestion	Reproducible, Cost effective, Removal of mass spectrometry incompatible detergents (SDS, Triton etc.) and contaminants, Wide cover range	Time consuming, Inacceptable for extremely acidic or basic and high or low molecular weight proteins and membrane proteins
In-sol Digestion	Require less time	Inacceptable for low resolubilization proteins
FASP	Acceptable for membrane proteins, Removal of mass spectrometry incompatible detergents (SDS, Triton etc.)	Loss of proteins , Bad repoducibility, Require large amount of protein sample (> 50ug)

Cho (2015) et al., suggested that the biggest issues in exosome research arise from the exosome isolation method. Since the proper isolation method for exosome study remains debated however, we summarized exosome isolation and exosomal protein digestion methods from the studies for exome proteome analyses in Table 2.

**Table 2.** Summarization of used techniques for cancer-derived exosomes isolation and exosomal protein digestion methods

Origin of Exosome	Isolation Method	Digestion Method	Reference
Cell	Ultracentrifuge	In-Gel Digestion	[23–30]
		In-Sol Digestion	[26, 31, 32]
	Precipitation reagent	In-Gel Digestion	[33–37]
		FASP	[38, 39]
	Combined method	In-Gel Digestion	[28, 35, 36, 40–51]
		In-Sol Digestion	[52–54]
		FASP	[51, 55–59]
Plasma/ Serum	Ultracentrifuge	In-Sol Digestion	[60]
		FASP	[56]
	Combined method	In-Gel Digestion	[43]
		In-Sol Digestion	[44, 52]
		FASP	[51, 57, 58]

The exosomes secreted from the cells and biological fluids are most often separated by a combined method. The most commonly used method is the fusion of ultracentrifugation and filtration. Exosomes are usually digested by the In-Gel , In- sol and FASP methods. Before the FASP method arose [61], the most used method was the In-Gel method. But the FASP method is known to have both the In-Gel and In-Sol methods’ advantages, thus recently manystudies used the FASP method for digestion regardless of where exosomes came from.

## Cancer-derived exosomal Proteins

### Breast cancer

Breast cancer is the deadliest cancer in women. One in eight women are diagnosed with breast cancer in their lifetime [62] and breast cancer accounts for 30% of newly diagnosed cancers in women [63]. For the last 10 years breast cancers’ death rates and incidence rates in The United States have risen each year. The exosome has been revealed as a potential liquid biopsy biomarker and numerous studies using liquid chromatography-mass spectrometry (LC-ms/ms) have revealed that cancer derived exosomes contains various proteins, including enzymes, membrane proteins, heat shock proteins, and even transcription factors.

There are many studies on different types of breast cancer exosomes; the cell line derived exosomes described, or exosomes derived from breast cancer patient biological fluids. An early exosome proteome study identified that exosomes derived from breast cancer cell lines MCF-7 and MDA-MB-231 have 59 and 88 proteins, respectively [64]. The MDA-MB-231 derived exosome contained more enzymatic proteins than the MCF7 derived exosome. The number of common proteins between the two cell lines are 27. These include cytoskeleton proteins such as β-actin, tubulin-β, and integrins; membrane proteins like BASP1; enzymes including enolase α and PRDX1; ribosomal proteins like RS27A; heat shock proteins including HSP90A, HSP90B, HSP7C; and epigenetic modification related proteins such as Histone proteins and 14-3-3 proteins. β-actin and tubulin-β are associated with breast cancer metastasis [65, 66]. Overexpression of these proteins in breast cancers show high metastatic potential. It has already been demonstrated by Hoshino et al that exosomal integrins α6/β4 and α6/β1 were related to lung metastasis and integrin αv/β5 was related to liver metastasis [32]. They also found that exosomal integrins activate the Src signaling pathway in the recipient cells, which induces the inflammatory response. There is no study yet regarding the correlation of BASP1 and breast cancer. But there is a study demonstrating that BASP1 overexpression promotes cervical cancer cell progression and can be a prognostic marker [67]. Enolase α is the glycolytic enzyme that catalyzes fructose-1,6-biphosphate to glyceraldehyde 3-phosphate and dihydroxyacetone phosphate. Research has revealed that an increased level of enolase α is related to breast cancer metastasis and drug resistance [68, 69]. PRDX1 is an antioxidant enzyme, but its role in breast cancer is controversial. It is, however, clearly overexpressed in breast cancer tissue relative to normal tissue [70]. Recently, Bajor et al demonstrated that PRDX1 is involved in reducing exogenous oxidative stress and induces cell growth in breast cancer [71]. The H2B1 (Histone H2B type 1-C/3/F/G/I) proteins are related to epigenetic regulation. Exosomal histone proteins have been detected in cancers and other diseases, and even in normal conditions [72, 73]. However, there are quantitative differences between those detected in cancer versus normal conditions. The role of exosomal histone proteins in recipient cells is currently controversial, but exosomes also contain 14-3-3 protein, which has been shown to bind with histone proteins [74, 75]. So, H2B1 can potentially induce epigenetic changes in recipient cells by binding with exosomal 14-3-3 proteins.

The most abundant MCF-7 derived exosomal proteins are the structural proteins such as fibronectin, annexin A1, vimentin, actin α, etc., and heat shock proteins. Fibronectin is also known to induce tumor progression and metastasis. The amount of fibronectin is much higher in the exosome of breast cancer patients’ plasma than normal plasma exosome [76]. A study revealed that fibronectin secreted by myeloma cell is attached to the recipient cell membrane and turns on p38 and pERK signaling [77]. Activated p38 and pERK signaling induces myeloma cell progression by activating DKK1 and MMP-9. The second largest presence after fibronectin is annexin A1, which is attached to the phospholipid membrane. Annexin A1 inhibits phospholipase A2 and induces anti-inflammatory activity [78]. Similarly, it is suggested that annexin A1 induces metastasis, macrophage polarization, and poor prognosis [79]. This provides support for Okano et al’s claim that increase in the amount of annexin A1 results in cell invasion which progresses into metastasis [80]. 5’-NTD (5’- nucleosidase, CD73) is the next dominant protein in the exosome of MCF7 cells. 5’-NTD is the enzyme that catalyzes the carbon 5’-nucleoside phosphorolytic cleavage and thus is essential for recycling adenosine and cell growth [81]. It is also overexpressed in many breast cancers. When 5’-NTD is overexpressed in breast cancer cells, it accelerated adhesion, migration and invasion of cancer cells [81–84]. So, it can be a clue for how MCF7 changes it microenvironment. That is cancer cells secretes a metastasis accelerator via the exosome. The 5’-NTD is also related to the immune response [85]. Exosomal 5’-NTD produces adenosine and indirectly modulates regulatory T cell (T_reg_) mediated immunity. Immune modulation for T_reg_ by 5’-NTD in cancer microenvironment also helps cancer cells for their growth and metastasis. Among the 59 proteins identified in the exosome secreted by MCF7, 30 proteins have been known to participate in breast cancer growth, metastasis, and chemoresistance.

The most abundant MDA-MD-231 derived exosomal protein is β-actin, which is frequently used as a housekeeping gene in the exosome. There are also other structural proteins in the exosome such as tubulin-β and keratins. In general, these cytoskeletal proteins of β-actin, tubulin-β and keratin are all associated with breast cancer metastasis. Fourteen other exosomal proteins are also known to be involved in the metastasis of breast cancer [65, 66, 86]. Tubulin-β is also known to induce chemotherapy resistance [87]. Palazzolo et al identified 32 proteins that are more abundant in the MDA-MB-231 exosome than MCF7 cells [88]. Of these 32 proteins, 5 overlap with exosomal proteins that Kruger et al identified. These 5 proteins, 14-3-3 protein epsilon, β-actin, annexin A1/5, heat shock protein 71 and galectin 3 binding protein can be potential biomarker candidates for breast cancer-derived exosome. In addition, a study also suggested del-1 as an early stage breast cancer exosome biomarker [89].

Klinke et al identified 27 and 28 proteins from other breast cancer cell lines, BT-474 and SKBR-3, respectively by secretome profiling through LC-ms/ms [90]. Some common proteins with MCF7 and MDA-MB-231 -derived exosomal proteins emerged, for example, β-actin, heat shock proteins, aldolase α, enolase α, 14-3-3 proteins, etc. BT-474 and SKBR-3 are HER2 positive cell lines, and secreted exosomes highly enriched with proteins involved in antigen presenting (HSPA5, CALR, PSME1,2, PSMA 3,6, PSMB 2,4, and HLA-C) and glycolytic metabolism (G6PD, TP1, and PGAM1). These proteins could lead to cancer immune-surveillance and malfunctioned energy synthesis in breast cancer microenvironment [91–93]. In addition, BT-474-derived exosomal proteins have a strong relation with neutrophil GO terms; DDX3X, VCP, HSP90AA1, ILF2, HSPA8, PNP, MME, MME2, PAB37, SERPINB6, GDI2, ALDOA, PGAM1, and GPI, which are related with neutrophil degranulation, mediated immunity and neutrophil activation. Immune suppression by cancer exosomes and its relation to neutrophils have already been studied [94]. In a breast cancer-bearing mice model, neutrophils were activated and exosome levels in blood were much higher than the normal control group. It is also suggested that exosomes derived from tumors interact with neutrophils and induce cancer-associated thrombosis. All these evidences strongly suggest the importance of cancer-derived exosome in the immune modulation.

In addition, exosomes are thought to help increase breast cancer tumorigenesis. This is due to the specificity of the proteins found in the exosomes. Firstly, the exosomes isolated from the serum of breast cancer patients had high amounts of survivin [95], a protein that controls anti-apoptosis of the surrounding cells, and exosomes isolated from the cell line had large amounts of MTA1 [96], a protein that promotes proliferation, . Secondly, there is a large amount of drug and chemoresistance proteins in the exosome. GSTP1, TGF-β1, TPRC5, and UCH-L1 are examples [97–100]. Finally, proteins that induce metastasis present highly in exosomes. A typical example is nephronectin, which has been reported to be high in the serum of patients with metastases [101]. It is also notable that analysis of breast cancer cells and their metastasized cancer cell derived exosomes revealed that integrin α6/β4, caveolin-1, periostin and myoferlin are more enriched in metastasized cancer cells than primary breast cancer cells [31, 32, 36, 102], although their roles need to be further investigated. These proteins might serve as biomarker candidates for breast cancer.

### Lung Cancer

Lung cancer is the most common cause of cancer related death in both sexes and also shows the highest incidence rate among cancer in the United States [63]. Non-small cell lung cancer (NSCLC) accounts for the largest proportion of lung cancer patients. This is further categorized into adenocarcinoma, squamous cell carcinoma, and large cell carcinoma. Lung cancer is found later than any other cancer because it has no symptoms that can be discerned through self-awareness. Thus, regardless of the many developed treatments for lung cancer, after diagnosis it is often too late to treat. This leads to a poor 5-year survival rate and motivates the search for scanning biomarkers. Here, we classify and summarize the roles of the NSCLC-derived exosomal proteins.

Clark et al analyzed two of the NSCLC cell lines, A549 and HCC827 [51]. They normalized the LC-ms/ms data with normal lung cell line HBE3. The number of proteins that expressed twice more in A549 than normal HBE3 is 58. Mucin 5 AC and B proteins are highly enriched in A549 derived exosome. Mucin 5 AC and B are known to only be expressed in lung adenocarcinoma. Overexpression of these proteins leads to lung cancer relapse and metastasis [103, 104]. Furthermore, there are Annexin proteins, ADAM10, EGFR, integrin, JAK, and metabolism related enzymes found in the A549 derived exosome. Of the 58 proteins, 12 (20%) are correlated with neutrophil degranulation and neutrophil-mediated immunity (JUP, C3, VCP, CD44, etc.). The HCC827 exosome has 93 more proteins than the HBE3 derived exosome. The most abundant protein is desmoglein-2. It has been reported that desmoglein-2 is overexpressed in NSCLC tissues and induce NSCLC growth by regulating p27 and CDK2 [105]. The next most enriched protein is EGFR. Given that EGFR is an oncogene in lung cancer, this can be a powerful clue to explain the exosome’s extreme tumorigenicity role in the neighboring microenvironment [106]. HCC827 derived exosomes also contain several kinds of other proteins; Integrin, Annexin proteins, guanine nucleotide-binding proteins (GPCR), and 14-3-3 proteins. Like in the A549 derived exosomal proteins, there are also neutrophil-related proteins enriched in the HCC287 derived exosome as well (22.5%).

The exosomes secreted by lung cancer, like the exosomes secreted by breast cancer, are also involved in tumorigenesis. Most cancers are characterized by metastasis only to certain organs, called organotropic metastasis [107]. Recent studies suggest that exosomes are also involved in organotropic metastasis [32]. Integrin plays a very important role. Hoshino et al revealed that treatment of exosomes isolated from lung cancer cells redirects lung cancer cells to metastasize to bone [32]. Exosomal integrins α6β4 and α6β1 are involved in lung cancer organotropic metastasis. As such, proteins in exosomes can induce metastasis by reprogramming cells. Exosomal Leucine-Rich-Alpha2-Glycoprotein 1 (LRG1) secreted from lung cancer cells induces angiogenesis through TGF-beta signaling in recipient cells [108]. This exosomal protein is also found in exosomes isolated from urine in patients with lung cancer [109], which may serve as a good prognostic marker. Another example is T-cell immunoglobulin- mucin-domain-containing molecule 3 (Tim-3) and its ligand Galectin-9 (Gal-9). Tim3 and Gal-9 exhibit anti-tumor immune responses, by blocking Th1 type immune responses [110]. Both proteins were found to be higher in the plasma derived exosomes of lung squamous cell carcinoma patients than in lung adenocarcinoma patients [110]. It is not yet known why these two proteins are contained in the exosome. Yet Tim-3 and Gal-9, can be used as prognostic and diagnostic markers of lung cancers.

NY-SEO-1, EGFR, PLAP, and EpCam were high in exosomes isolated from the plasma of lung cancer patients [111], and Vykoukal et al also revealed that SGRN, TPM3, THBS1, and HUWE1 levels in plasma-derived exosomes in lung cancer patients is higher than control group [112]. EGFR is a protein that is highly related to lung cancer, and is abundant in exosomes isolated from lung cancer cells, lung biopsies and plasma, thus making it the most powerful biomarker from the revealed candidates [111, 113, 114]. Exosomes derived from NCI-H838, another NSCLC cell line, contain more MUC1 as revealed in patients’ plasma exosomes. Other report also revealed that MUC1 in NSCLC patient plasma exosome as much higher than that in the normal control group plasma exosome [115].

### Other cancers

Among all women and men diagnosed with cancer each year, there is a high proportion of people diagnosed with colon cancer [63]. Colon cancer cell derived exosomal proteins are identified by Choi et al [42] and Mathivanan et el [33]. The common proteins of both studies are related with cancer progression and metastasis. Choi et al also suggested that the identified proteins have relation with immune modulation. Furthermore, human colon cancer ascites derived exosomes have similar tumorigenesis potential with proteins related to cancer progression, immune modulation, and metastasis [116]. Not only ascites, but also serum exosome can be a good diagnostic marker. Annexin proteins, and tspan 1 from the serum exosome are suggested colon cancer diagnostic markers [35, 117]. However, most cancer derived exosomes, as mentioned in breast and lung cancer derived exosomes as above, contain a lot more annexin and tetraspanin proteins than normal control derived exosome. So, these two proteins rather are pan-cancer exosome proteins.

Pancreatic cancer incidence and death rate have been increasing. Moreover, the 5-year relative survival rates of pancreatic cancer is only 9%, whereas other cancers such as prostate is 98% and melanoma is 92% [63]. Pancreatic cancer cell derived exosome that induce metastasis [118, 119] and chemoresistance [27] have been revealed. Several proteins, glypican-1, CD44, Tspan8, EpCam, MET and CD104, have been suggested as pancreatic cancer exosome-derived biomarkers [31, 120]..

Renal cancer incidence rate is 3 to 5 % for both males and females, but there is no accurate biomarker for renal cancer. Raimondo et al identified renal cancer patients’ urinary exosomes [121]. They suggested 10 proteins for renal cancer exsome biomarker, 5 of which are abundant in renal cancer patients; matrix metalloproteinase 9 (MMP-9), ceruloplasmin (CP), podocalyxin (PODXL), dickkopf related protein 4 (DKK4) and carbonic anhydrase IX (CAIX). Oppositely, aquaporin-1 (AQP1), extracellular Matrix metalloproteinase Inducer (EMMPRIN), neprilysin (CD10), dipeptidase 1 and syntenin-1 are abundant in the exosome of healthy control. They claimed that these 10 proteins have great potential for early stage diagnosis of renal cancer with clinical value. A summary of selected exosomal proteins is given in Table 3.

**Table 3:** Description of the selected exosomal proteins in cancer

Exosomal Protein	Description
Breast Cancer	
β-actin	Breast cancer metastasis [65]
Tubulin-β	Breast cancer metastasis, chemotheray resistance [66, 87]
Integrin α6/β4, α6/β1	Lung metastasis [32]
Integrinαv/β5	Liver metastasis [32]
BASP1	Overexpression leads to ovarian cancer cell progression [67]
Enolase A	Breast cancer metastasis and drug resistance [68, 69]
PRDX1	Induce cell growth in breast cancer and overexpressed in breast cancer [70]
14-3-3 protein	Bind to histone protiens and induce epigenetic changes [75]
Fibronectin	Tumor progression and metastasis [76]
Annexin A1	Induce tumor metastasis and macrophage polarization [78–80]
5’-NTD	Overexpressed in breast cancer cells and induce metastasis [81–85]
Survivin	Overexpressed in breast cancer serum derived exoxome, Anti apoptosis [95]
MTA1	Promote proliferation [96]
GSTP1	Drug and chemotherapy resistance [97]
TGF-β1	Drug resistance [98]
TPRC5	Chemotherapy resistance [99]
UCH-L1	Chemotherapy resistance [100]
Nephronectin	Induce tumor mestasis [101]
Caveolin-1	Enriched in metastasized cancer cell [36]
Periostin	Enriched in metastasized cancer cell [102]
Myoferlin	Enriched in metastasized cancer cell [31]
Lung Cancer	
Mucin 5AC, B	Lung cancer relapse and metastasis [103, 104]
Desmoglein-2	overspressed in non small cell lung cancer and induce cell growth [105]
EGFR	Oncogene in lung cancer [106, 111, 113, 114]
LRG1	Induce angiogenesis [108]
Tim-3	Induce anti-tumor immune response [110]
NY-SEO-1	Overexpressed in lung cancer [111]
PLAP	Overexpressed in lung cancer [111]
EpCam	Overexpressed in lung cancer [111]
SGRN	Overexpressed in lung cancer [112]
TPM3	Overexpressed in lung cancer [112]
THBS1	Overexpressed in lung cancer [112]
HUWE1	Overexpressed in lung cancer [112]
MUC 1	Overexpressed in lung cancer [112]
Colon Cancer	
Annexin family	Colon cancer progression and metastasis [33, 42]
Tetraspanin 1	Colon cancer progression and metastasis [33, 42]
Pancreatic Cancer	
Glypican-1	Abundant in pancreatic cancer exosome [31, 120]
CD44	Abundant in pancreatic cancer exosome [31, 120]
Tspan 8	Abundant in pancreatic cancer exosome [31, 120]
EpCam	Abundant in pancreatic cancer exosome [31, 120]
MET	Abundant in pancreatic cancer exosome [31, 120]
CD104	Abundant in pancreatic cancer exosome [31, 120]
Renal Cancer	
MMP-9	Abundant in renal cancer exosome [121]
CP	Abundant in renal cancer exosome [121]
PODXL	Abundant in renal cancer exosome [121]
DKK4	Abundant in renal cancer exosome [121]
CAIX	Abundant in renal cancer exosome [121]
AQP1	Abundant in renal cancer exosome [121]
EMMPRIN	Abundant in renal cancer exosome [121]
CD10	Abundant in renal cancer exosome [121]
Dipeptidase 1	Abundant in renal cancer exosome [121]
Syntenin-1	Abundant in renal cancer exosome [121]

## The role of exosome proteins in tumor microenvironment: Friend or Foe ?

### Tumor microenvironment

Many approaches to conquer cancer are ongoing all over the world. Nevertheless, incidence and death rates of cancer are on the rise every year [63]. The main cause of the increasing incidence rate and mortality is not only primary tumors, but also distant tumors [122]. Many researchers have been trying to understand the mechanism of metastasis to find a cure the cancer [123–125]. A rising concept for the metastasis mechanism is that the tumor is collaborating with the tumor microenvironment through exosomes.

**Fig. 3:**
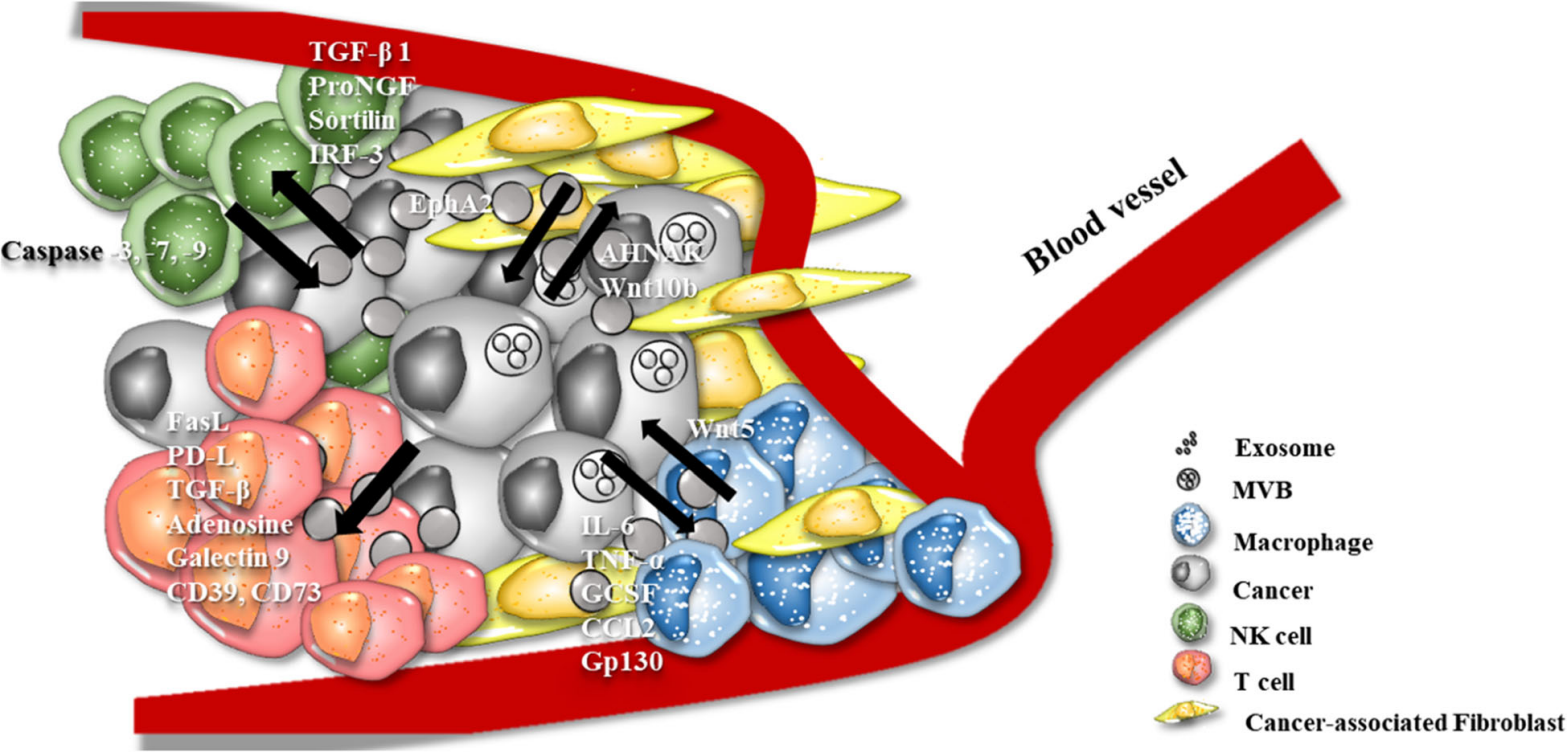
The function of cancer and other components of tumor microenvironment-derived exosome. Cancer-derived exosomes contain various types of proteins for immune suppression, cancer progression and metastasis

The tumor microenvironment consists of immune cells, fibroblasts, the extracellular matrix, basement membrane, endothelial cells, and cancer cells [126–129]. Several roles of the tumor microenvironment have been suggested [126]. Component cells of the tumor microenvironment have roles in tumor initiation, progression, and metastasis. Many studies have revealed that the components of the tumor microenvironment communicate via exosomes [1, 130–134]. Here, we summarized potential roles of the exosome between cancer cells and tumor microenvironment cells, and the effect on tumorigenesis (Fig. 3).

### Cancer associated fibroblast (CAF)

A predominant stromal cell component of the tumor microenvironment is activated by a fibroblast, termed cancer-associated fibroblast (CAF) [135]. Several studies have revealed that CAFs are highly involved in tumor progression [136–138]. Mammary carcinoma-derived exosomes induce mammary fibroblast motility by transferring AHNAK [139]. As such, the exosome secreted by cancer cells can affect fibroblasts, but here we also discuss how fibroblast-derived exosome can affect cancer cells. Many researchers have suggested that CAFs have a relation to cancer proliferation, chemoresistance, and metastasis. Takasugi et al demonstrated that senescent fibroblast-derived exosome can induce MCF7 cell proliferation by transferring EphA2 [140]. In pancreatic cancer, it is reported that chemotherapy stimulated CAFs to release more exosomes, which in turn promoted recipient cancer epithelial cells’ proliferation and drug resistance [141]. Exosomes secreted by CAFs also induce epithelial-mesenchymal transition (EMT), migration, and invasion, resulting in metastasis and cell growth of bladder cancer by activating IL-6 signaling [142]. TGFβ1 is enriched in ovarian CAFs and affects ovarian cancer cells into EMT by SMAD signaling activation [143]. In lung cancer, CAF-derived exosome also enhance metastasis by activation of the IL-6/STAT3 signaling pathway [144]. Furthermore, exosomes secreted by CAFs can affect chemotherapy resistance [145]. It is suggested CAFs-derived exosome in colorectal cancer stem cells can promote drug resistance and enhance cancer stem cell properties and also growth.

### Natural killer (NK) cell

Natural killer (NK) cells are large granular cytotoxic lymphocytes that kill the target cancer cells without stimuli. There are several mechanisms by which NK cell-derived exosomes kill the recipient cells [146]. Activated NK cell-derived exosomes are cytotoxic because they can induce the cell death pathway by perforin (PFN), granzymes (Gzm-A/B) and granulysin (GNLY). Wen et al suggested that activated NK cell exosomes deliver caspase inducers which lead to cancer death by activation of the caspase-dependent cell death pathway. Perforin is delivered to form the pores and granzymes enter into the recipient cells. Granzymes induce caspase-dependent and -independent cell death. Cancer cells incubated with activated NK cells showed an increased expression of activated caspase-3, -7, and -9 [147]. Prostate cancer-derived exosomes downregulate NKG2D expression on NK cells and CD8+ T-cells. This leads to downregulation of NKG2D-mediated cytotoxic response in prostate cancer patients via immune escape [148]. Berchem et al showed that hypoxic tumor-derived exosomes transfer TGF-β1 to NK cells, which leads to the down regulation of NK cell’s surface expression of NKG2D receptor [149]. Decreasing NKG2D inhibits NK cell function. Renal cancer cell-derived exosomes are enriched with TGF-β1 and activate the TGF-β1/SMAD pathway in NK cells to facilitate immune escape [150]. Another mechanism of NK cell immune response escape is related to p75NTR. NK cells in tumor microenvironment have high expression of p75NTR and exosomes secreted by lung cancer contain proNGF and sortilin, which bind to p75NTR and induce NK cell apoptosis [151]. Interestingly, exosomes released from cancer cells do not always inhibit the activity of NK cells. Wang et al revealed that ovarian cancer cell-derived exosomes enhance the cytotoxicity effect of NK cells [152]. Exosomes secreted by ovarian cancer cell contain phosphorylated IRF-3 that promotes NK cell cytotoxicity by inducing interferon gene expression in NK cells.

### T-cell

Cancer-derived exosomes are known as immune suppressors because they can inactivate effector T cells and induce T cell apoptosis. There were a lot of research conducted on cancer-derived exosomes and T cell interaction. Cancer-derived exosomes could induce T cell suppression by delivering Fas ligand (FasL), PD-L1, TGF-β, adenosine and galectin-9. Abusamra et al demonstrated that prostate cancer cell-derived exosomes have FasL that induces T cell apoptosis upon delivery by caspase activation [153]. Colorectal cancer-derived exosomes have shown the same effect on T cell apoptosis [154]. The exosome isolated from head and neck cancer patients’ serum also induces T cell apoptosis [155]. Tumor cells expressing PD-L1 in their membrane can escape form the immune response. PD-L1 is also detected in cancer-derived exosomes. Prostate and melanoma cancer cell-derived exosomal PD-L1 binds to effector T cell’s membrane PD-1 receptor, thereby impairing their growth [156]. Melanoma patients’ circulating exosomes also have PD-L1, which also induces immune surveillance.

Exosomal TGF-β 1 is a well-known immune surveillance factor [157]. Hypoxia conditioned BT-474 and MDA-MB-231 secreted more exosomes than normoxia condition. These exosomes have an increase in TGF-β which inhibits T cell proliferation. Colorectal cancer-derived exosomes were enriched with TGF-β1, which induces alteration of T cell phenotype to T regulatory cells by activating TGF-β/Smad signaling and inactivating SAPK signaling [158]. Prostate cancer cell line-derived exosomes also contribute to immune evasion by transferring exosomal TGP-β1 [159].

Adenosine is also a key factor in a known pathway that induces T cell suppression. Tumor exosomes have been known to contain CD39 and CD73 on the membrane surface. Exosome-mediated transfer of CD39 and CD73 leads to hydrolysis of ATP to adenosine. Accumulated adenosines participate in T cell inactivation by binding with their receptors (A1, A2A, A2B, and A3) [160]. Adenosine binds their receptors in T_reg_ cells and triggers the cyclic AMP (cAMP) and protein kinase A (PKA) signal [161]. This signal can regulate either survival or apoptosis of the T cell, depending on the signal strength and duration [162].

Galectin also has a role in tumor-derived exosome induced immune escape. Galectin-1 is a type of β-galactosidase protein expressed in immune cells. Recently, it has been shown that tumors secreting galectin-1 on a high level affect immune cells by binding to N-acetyllactosamine on the T cell membrane. Interaction of galectin-1 and the galectin ligand induces immune escape through T cell apoptosis [163]. It is also demonstrated enrichment of galectin in head and neck cancer-derived exosome induced suppression of CD8^+^ T cell [164].

### Macrophage

Macrophages belong to the innate immune system. Macrophages are divided into two types by Th1 and Th2 polarization, which are called M1 and M2, and have the characteristics of pro-inflammatory and anti-inflammatory, respectively [165]. Tumor-associated macrophages (TAMs) consist of M2 characteristic macrophages and promote angiogenesis, invasion, and metastasis [166]. M2 macrophages secrete tumor metastasis supporting cytokines such as CCL2, MIP2, IL-8, and IL-Rα and attenuating antitumor cytokines such as TIMP-1, IFN-γ, IL-1Rα, IL-13, and IL-16 [167, 168]. Chen et al described how colorectal cancer-derived exosomes induce M2 macrophages by cytoskeleton rearrangement [169]. Gastric cancer-derived exosomes also have effect to M2 macrophage [170].

On the other hand, it has been also reported that gastric cancer-derived exosomes activate the NF-kB pathway in recipient macrophages, leading to up-regulation of pro-inflammatory factors [171] such as IL-6 and TNF-α which promote gastric cancer progression [172]. A similar result was also reported that breast cancer cell line-derived exosome stimulates the NF-kB pathway in macrophages, which leads to the secretion of pro-inflammatory factors such as IL-6, TNF-α, GCSF, and CCL2 [173]. Breast cancer exosomal protein HSP72 and RNAs are involved in stimulating the NK-kB pathway. It was also demonstrated that breast cancer-derived exosomes promote macrophage polarization and induce lymph node metastasis [174]. It has been also shown that breast cancer-derived exosomes are enriched with gp130, which induces gp130/STATS signaling in macrophage and leads to the secretion of IL-6 for macrophage polarization [175]. From this point of view, tumor-derived exosomes are assumed to play both anti-inflammatory and pro-inflammatory roles by stimulating M2 and M1 macrophages, respectively.

TAM-derived exosomes also participate in tumorigenesis. For example, TAM-derived exosomes enhance tumor invasion by delivering wnt5a in macrophages to breast cancer cells. Delivered wnt5a enhances tumor invasion by leading to the activation of β-catenin-independent Wnt signaling [176].

## Conclusion

Since exosomes are known as cellular communicators, there are many approaches for isolation and exosomal content analysis. Sadly, the big hurdles of exosome research still remain. The establishment of isolation and digestion standards remain essential. In this review, we briefly summarized the current methods of exosome isolation and its protein digestion. For cell supernatant exosome analysis, exosomes are isolated by combined methods and proteins are digested mostly by In-Gel digestion and FASP. Body fluid exosomes are isolated by the same methods, but proteins are digested by FASP for mass spectrometry.

Cancer-derived exosomes contain various proteins. Exosomal proteins from cancer cells affect the tumor microenvironment in their favor through suppressive modulation of immune cells including NK cells, T cells, and Macrophages and immune surveillance. Cancer stem cell progression and chemotherapy resistance are acquired by modulating cancer associated fibroblasts. Together, cancer-derived exosomes and tumor microenvironment cell-derived exosomes alter the cancers to be more aggressive and be able to metastasize. In the process, these tumor-derived exosomes are enriched and can be detected in biological fluids. Recent discoveries in exosome fields will also alter cancer management. Indeed, non-invasive diagnosis and prognosis could become possible via plasma exosomes. Thus, it is suggested that strategies based on the blocking of exosomal immune suppression could be developed for the treatment of cancer patients. Since exosome fields are expanding, further efforts to reveal fundamental mechanisms of exosome cargo selection and biogenesis are necessary to fully understand the roles of proteins in exosomes.

## Data Availability

Not applicable.
